# Treatment Patterns and Outcomes of Patients With Human Epidermal Growth Factor Receptor 2–Positive Breast Cancer in Tanzania

**DOI:** 10.1200/GO-24-00612

**Published:** 2025-08-07

**Authors:** Lemi Ndugumbi Zinga, Queen Godfrey Tarimo, Kelvin Mbelekwa, Mamsau Twalib Ngoma, Emmanuel L. Lugina

**Affiliations:** ^1^ Muhimbili University of Health and Allied Sciences (MUHAS), Dar es Salaam, Tanzania; ^2^ Ocean Road Cancer Institute (ORC), Dar es Salaam, Tanzania

## Abstract

**PURPOSE:**

Human epidermal growth factor receptor 2 (HER2)–positive breast cancer (HER2+BC) is linked to poorer outcomes. Trastuzumab is the standard treatment, but its high cost limits access in resource-limited settings. This study evaluated the treatment patterns and survival outcomes of patients with HER2 BC in Tanzania.

**MATERIALS AND METHODS:**

A retrospective hospital-based study was conducted between January 2018 and May 2021 at two prominent public tertiary hospitals in Tanzania. The primary outcome was the 5-year overall survival (OS) rate. Independent variables included demographics, clinical characteristics, and treatment patterns. Survival analysis was conducted using the Kaplan-Meier method. The log-rank test was used in univariate analysis, whereas multivariate Cox regression was used in multivariate analysis.

**RESULTS:**

A total of 169 patients with nonmetastatic HER2+BC were included, with a median age of 50 years. Most patients were estrogen receptor–positive (50.3%) and diagnosed with stage III BC (58.6%). About 70.4% of patients received trastuzumab, whereas neoadjuvant chemotherapy was administered to 24.3%, and 95.9% underwent mastectomy. Trastuzumab was primarily used in the adjuvant setting rather than the neoadjuvant setting and was more accessible to patients with higher socioeconomic status. The 5-year OS was 65%, with a median survival of 72 months. Trastuzumab reduced the mortality risk by 74%. Extended therapy with trastuzumab benefited patients with hormone receptor–negative HER2+BC more than those who were hormone receptor–positive. Advanced-stage disease increased mortality risk, whereas postmenopausal status and nonsmoking were associated with lower mortality. A higher Eastern Cooperative Oncology Group score (>1) was associated with an increased mortality risk.

**CONCLUSION:**

Trastuzumab significantly improves OS in resource-limited settings, particularly in patients with hormone receptor–negative HER2+BC. There are disparities in access to trastuzumab in Tanzania.

## INTRODUCTION

Breast cancer (BC) accounts for 25% of all cancer cases and 15% of cancer-related mortality among women worldwide, making it the leading cause of cancer-related deaths in this group.^1,2^ The global cancer burden is projected to rise by 47% from 2020 to 2040,^3,4^ with sub-Saharan Africa (SSA) expected to experience a doubling in BC prevalence by 2050.^3^ Despite low- and middle-income countries (LMICs) bearing almost 80% of the burden, measured by disability-adjusted life years, they receive <5% of global cancer-fighting resources.^3^

**CONTEXT**

**Key Objective**
The objective of this study was to assess the treatment patterns and survival outcomes of patients diagnosed with human epidermal growth factor receptor 2 (HER2)–positive breast cancer (HER2+BC) in Tanzania.
**Knowledge Generated**
This study revealed that 70.4% of patients received trastuzumab, predominantly in the adjuvant setting; however, access was skewed toward those with higher socioeconomic status. The 5-year overall survival was 65%. The use of trastuzumab reduced the mortality risk by 74%. The study highlighted that extended trastuzumab therapy, particularly for patients with hormone receptor–negative HER2+ breast cancer, benefited them significantly. Advanced-stage disease and higher Eastern Cooperative Oncology Group scores were associated with an increase in mortality risk, whereas postmenopausal status and nonsmoking were associated with improved survival outcomes.
**Relevance**
The study's findings indicate disparities in access to trastuzumab in Tanzania. This study underscores the pressing need to expand access to this medication in resource-limited settings, which would significantly affect survival.

BC is the most common malignancy, causing high mortality among women globally.^4^ It is genetically and clinically heterogeneous, with multiple subtypes. Routine immunohistochemistry (IHC) parameters classify BC into four molecular subtypes based on hormone receptor expression: estrogen receptor–positive (ER+), progesterone receptor–positive (PR+), human epidermal growth factor receptor 2–positive (HER2+), and triple-negative breast cancer, which lacks all these receptors.^5,6^ The subtypes include luminal A (ER+ and/or PR+/HER2–/low Ki-67), luminal B (ER+ and/or PR+/HER2–/high Ki-67), HER2-positive luminal B (ER+ and/or PR+/HER2+/any Ki-67), nonluminal HER2+ (ER– and PR–/HER2+), and triple-negative (ER– and PR–/HER2–).^5,6^

HER2+BC, which represents 15%-20% of cases globally, is aggressive, with high mortality rates and poor responses to conventional chemotherapy.^7,8^ Anti-HER2 therapies, particularly trastuzumab, have transformed treatment paradigms, improving survival and cure rates in patients with HER2+BC.^1^ Trastuzumab, combined with chemotherapy, is recommended in Tanzania's national and international guidelines for HER2+BC and has been on the WHO's essential medicines list since 2015.^9^ However, access to trastuzumab remains limited in LMICs, affecting treatment outcomes.^9^

Access to trastuzumab and other treatments remains inconsistent in LMICs, which affects treatment patterns and patient outcomes.^9^ In Tanzania, where <15% of the population is fully insured, many patients with cancer face financial barriers to receiving optimal treatments^10^ because of out-of-pocket payments. This situation affects cancer outcomes as patients often cannot afford the recommended first-line therapies, including targeted HER2 treatments.^11,12^

Although data exist on the treatment and outcomes of HER2+BC in high-income countries, there are limited local data on treatment patterns and outcomes in Tanzania. This study aimed to assess treatment patterns for HER2+BC in a low-income setting and the outcomes related to targeted HER2 therapies.

## MATERIALS AND METHODS

### Study Framework and Data Extraction

The study was conducted using a retrospective cohort design. Data on sociodemographics, clinical presentation, histopathology, treatment (eg, surgery, chemotherapy, radiotherapy), and long-term outcomes of patients with HER2+BC treated from January 2018 to May 2020 were extracted from the electronic medical records of patients with HER2+BC at two prominent public tertiary hospitals in Dar es Salaam, Tanzania: The Ocean Road Cancer Institute (ORCI) and Muhimbili National Hospital (MNH). The primary outcome was the 5-year overall survival (OS) rate.

### Study Population

The study population consisted of patients 18 years or older who were diagnosed with stage II and III HER2+BC, confirmed through histologic assessments and IHC techniques, including HER2/neu IHC 3+, fluorescence in situ hybridization, or in situ hybridization. Patients were excluded if they had distant metastatic disease at diagnosis, if their treatment regimens were not documented, if they had other primary malignancies, or if they presented with cardiac contraindications to HER2-targeted therapy.

### Statistical Analysis

Statistical analysis was performed using R version 2024.12.0+467. The multiple imputation method was used to replace missing data when the percentage of missing data in a variable was <10%. Variables with a percentage of missing data exceeding 10% were deleted. Descriptive statistics were used to summarize the characteristics of the study population, with categorical variables reported as frequencies and percentages and continuous variables expressed as medians and interquartile ranges. The categorical variables were compared using the chi-square test. The continuous variables were compared using the student’s *t* test. The Kaplan-Meier method was used to estimate survival curves. A log-rank test was used in the univariate analysis, and a Cox proportional hazards regression model was used in the multivariate analysis. The Schoenfeld residual test was used to assess whether the proportional hazards assumptions were met. When Cox's proportional hazard assumptions were violated, modeling time-varying coefficients in the Cox model was used. A *P* value of <.05 was considered statistically significant.

### Institutional Review Board Approval and Ethical Considerations

The Institutional Review Board of the Muhimbili University of Health and Allied Sciences (MUHAS) granted ethical approval for this study. In addition, permissions were obtained from MNH and ORCI's respective ethics committees to conduct the research. Because of the study's retrospective nature, a waiver of informed consent was approved by the MUHAS IRB, acknowledging the minimal risk involved. Strict confidentiality protocols were maintained throughout the research to ensure patient anonymity, with no personally identifiable information disclosed. Verbal consent was sought from patients or their next of kin during follow-up communications to verify their current health status, ensuring ethical compliance and respect for patient autonomy.

## RESULTS

Of 1,831 patients, 169 patients with stage II and III HER2 BC who met the eligibility criteria were included in the study.

The median age was 51 years, with an even split between those 50 years or younger (48.5%) and those 51 years or older (51.5%). Trastuzumab was administered to 85.7% of patients with education beyond secondary school, compared with 59.7% of those with secondary education or less. The impact of employment status was similar: 89% of employed or retired patients received trastuzumab, whereas only 57.7% of peasants or unemployed patients received it. Health insurance played a substantial role as 86.7% of insured patients received trastuzumab compared with 44.6% of patients paying out of pocket. Hypertension (36.1%) was the most common comorbidity. Invasive ductal carcinoma was the dominant histologic type (92.3%), and approximately 50% was ER+. Most patients had stage 3 BC (58.6%). Most patients with an Eastern Cooperative Oncology Group (ECOG) score of 0 received trastuzumab (81.7%), compared with those with an ECOG status of 1 or higher (55.9%; *P* = .00). About 70.4% of patients received at least one cycle of trastuzumab, averaging 10.2 cycles (median: 9, range, 1-18). Neoadjuvant chemotherapy (NACT) was administered to 41 patients (24.3%), with 31% receiving adriamicin, cyclophosphamide-taxane regimen (AC-T) and 28.5% receiving taxane adriamicin cyclophosphamide regimen. Surgical intervention was offered to 162 patients (95.9%), and 115 patients (68.1%) underwent chest wall radiotherapy, primarily via 3DCRT. Adjuvant chemotherapy was administered to 145 patients (85.8%), with the AC-T regimen being the most commonly used (69.2%). Trastuzumab was administered more frequently with adjuvant than with NACT (69.0% *v* 41.7%, *P* < .001; Table 1).

**TABLE 1:** Characteristics of Patients

Characteristic	Total Cohort (N = 169), No. (%)	Trastuzumab Given, No. (%)		*P*
		Yes	No	
Age, years				
≤50	82 (48.5)	52 (63.4)	30 (36.6)	.66
>50	87 (51.5)	58 (66.7)	29 (33.3)	
Menopausal status				
Premenopausal	62 (36.7)	38 (61.3)	24 (38.7)	.43
Postmenopausal	107 (63.3)	72 (67.3)	35 (32.7)	
Education level				
Below secondary	134 (79.3)	80 (59.7)	54 (40.3)	.04
Beyond secondary	35 (20.7)	30 (85.7)	5 (14.3)	
Occupation				
Retired/employed	39 (23.1)	35 (89.7)	4 (10.3)	.00
Peasant/unemployed/self-employed	130 (76.9)	75 (57.7)	55 (42.3)	
Smoking				
Yes	2 (1.2)	1 (50.0)	1 (50.0)	.65
No	162 (98.2)	109 (65.3)	58 (34.7)	
Payment mode				
Health insurance	83 (49.1)	72 (86.7)	11 (13.3)	
Out of pocket	86 (50.6)	38 (44.2)	48 (55.8)	.00
Hypertension				
Yes	61 (36.1)	41 (62.7)	20 (32.8)	
No	108 (63.9)	69 (63.9)	39 (36.1)	.66
Stage				
II	70 (41.4)	49 (70.0)	21 (30.0)	.26
III	99 (58.6)	61 (61.6)	38 (38.4)	
ECOG				
0	60 (35.5)	49 (81.7)	11 (18.3)	.001
≥1	109 (64.5)	61 (55.9)	48 (44.0)	
Neoadjuvant chemotherapy				
Yes	41 (24.3)	21 (51.2)	20 (48.8)	.03
No	128 (75.7)	89 (69.5)	39 (30.5)	
Adjuvant chemotherapy				
Yes	145 (85.8)	100 (69.0)	45 (31.0)	.009
No	24 (14.2)	10 (41.7)	14 (58.3)	
Hormonal therapy				
Yes	72 (42.6)	51 (70.8)	21 (29.2)	.12
No	97 (57.4)	59 (60.8)	38 (39.2)	

Abbreviation: ECOG, Eastern Cooperative Oncology Group.

**FIG 1:**
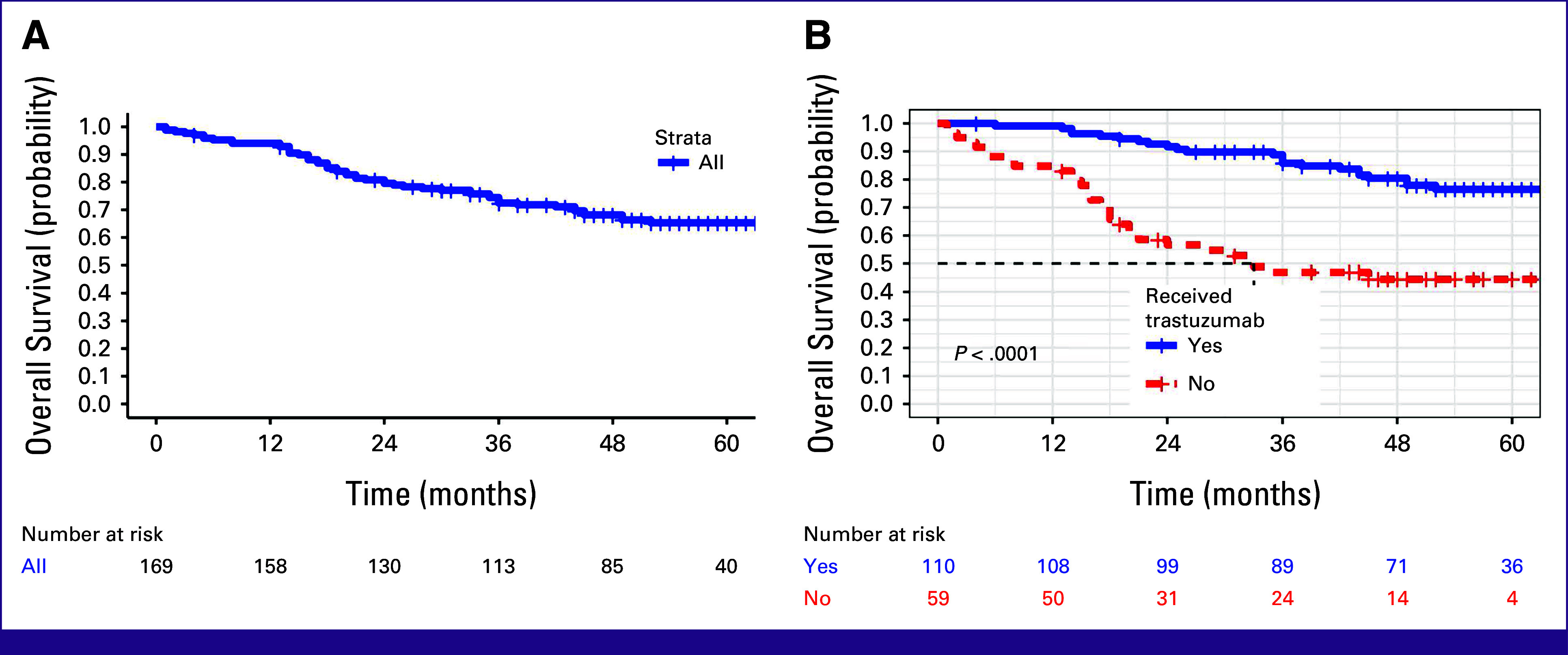
(A) The Kaplan-Meier overall survival curve for patients with HER2+BC and (B) Kaplan-Meier survival curve for patients with HER2+BC by their trastuzumab status (N = 169). BC, breast cancer; HER2, human epidermal growth factor receptor 2.

The 5-year OS rate was 65%, with a median OS time of 72 months (Fig 1A).

Patients who received trastuzumab experienced a higher 5-year OS rate compared with those who did not receive the treatment (*P* < .001; Fig 1B).

**FIG 2:**
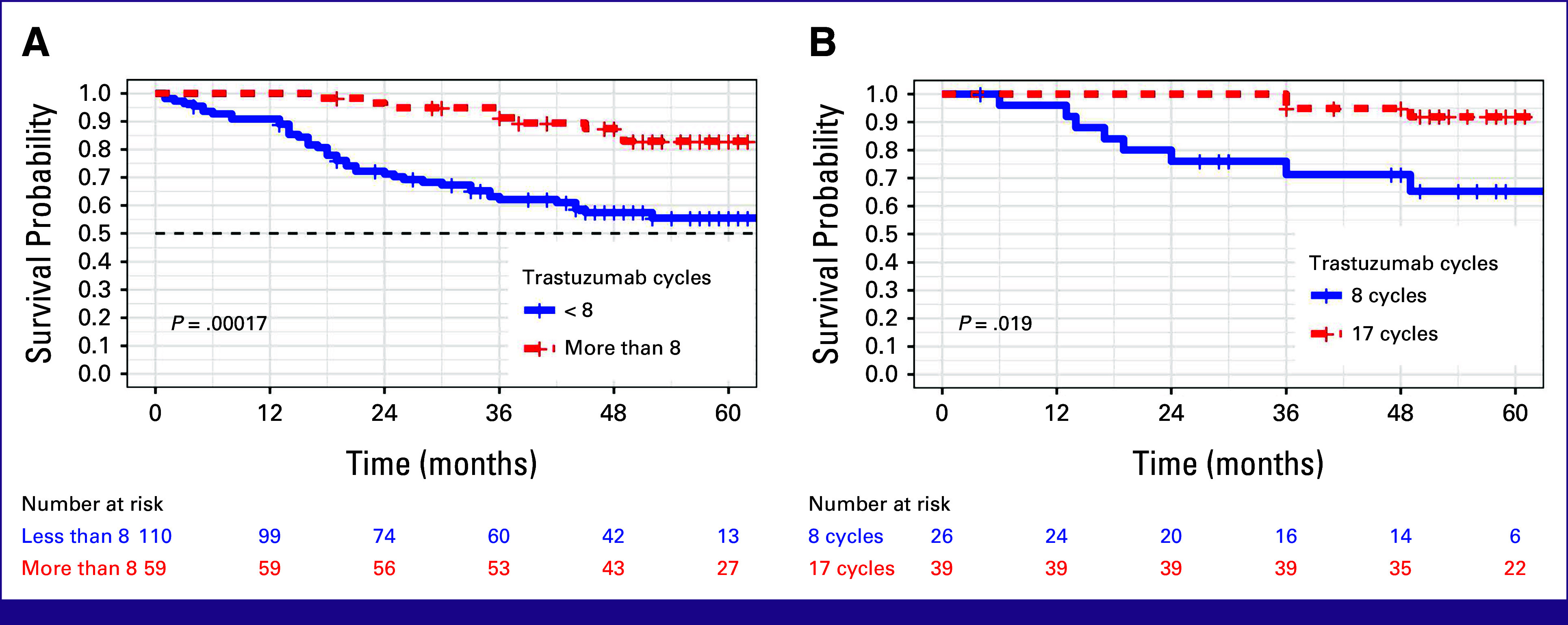
(A) Kaplan-Meier survival curve for patients with HER2+ BC who received more than eight cycles and those who received fewer than eight cycles of trastuzumab (N = 169) and (B) subgroup analysis comparing patients who completed eight cycles of trastuzumab with those who completed 17 cycles (n = 65). BC, breast cancer; HER2, human epidermal growth factor receptor 2.

Patients who received more than eight cycles of trastuzumab exhibited a higher 5-year OS rate than those who received fewer than eight cycles (*P* value <.001; Fig 2A).

The results indicate that patients who completed 17 cycles had a higher 5-year OS rate compared with those who completed only eight cycles (*P* value = .019; Fig 2B).

**FIG 3:**
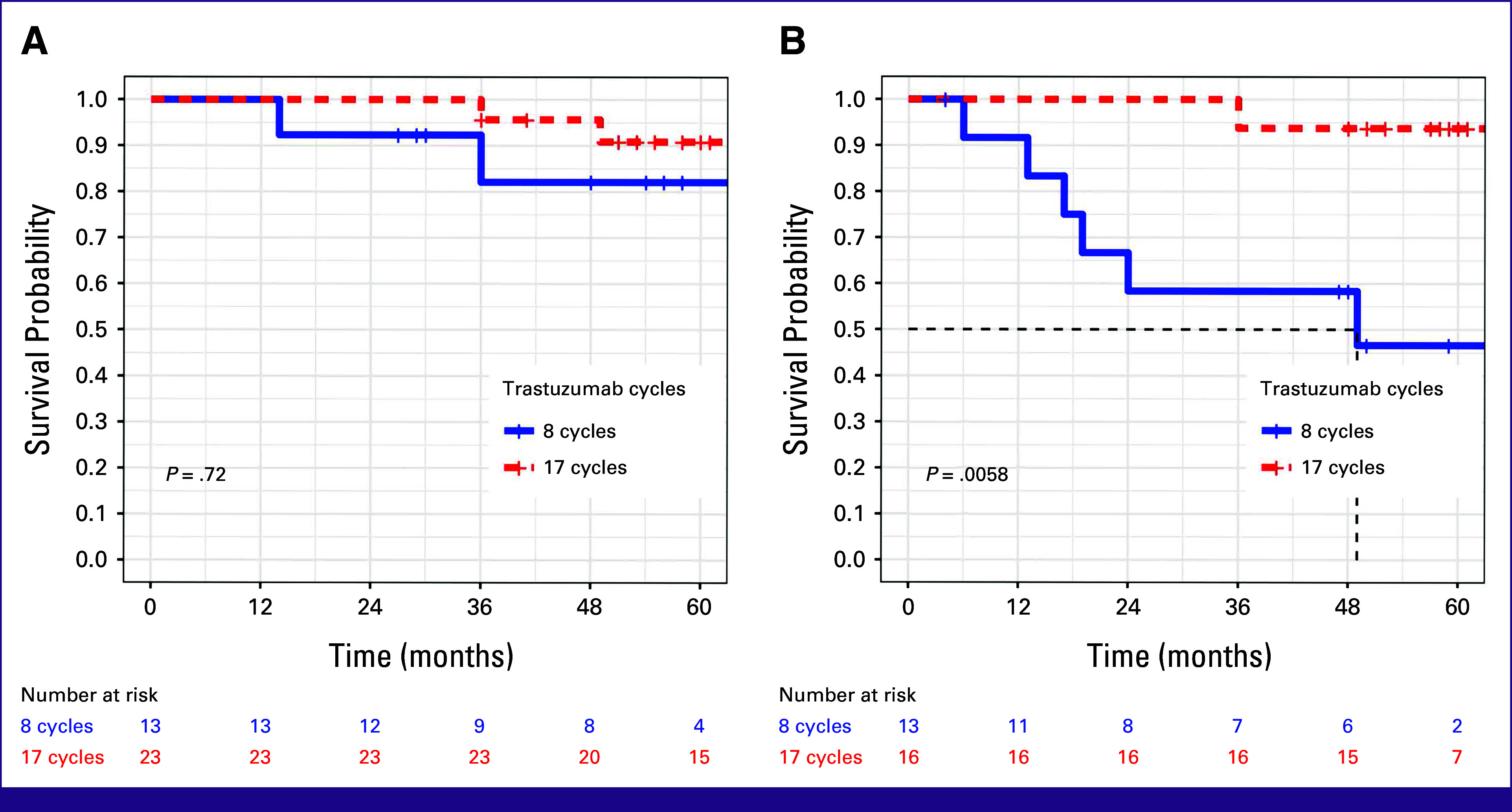
(A) Subgroup analysis comparing patients with ER-positive HER2+BC who completed eight cycles of trastuzumab with those who completed 17 cycles (n = 36) and (B) Subgroup analysis comparing patients with ER-negative HER2+BC who completed eight cycles with those who completed 17 cycles of trastuzumab (n = 29). BC, breast cancer; ER, estrogen receptor; HER2, human epidermal growth factor receptor 2.

The findings reveal no significant difference in OS rates between patients with ER+HER2+BC who had eight cycles of trastuzumab and those who had 17 cycles (*P* value = .2; Fig 3A).

Patients with ER-HER2+BC who completed 17 cycles had a higher 5-year OS rate than those who completed only eight cycles (*P* value = .04; Fig 3B).

Factors that were significantly associated with increased mortality were premenopausal status, having relapsed, hypertension, an ECOG status of more than 1, smoking, advanced stages, lack of health insurance, not having been given trastuzumab, and not having been given adjuvant chemotherapy (Table 2).

**TABLE 2:** A Summary of Univariate Analysis

Variable	Univariate Analysis		
	*P*	cHR	95% CI
Menopausal status			
Premenopausal	.006	2.4	1.3 to 4.6
Postmenopausal	1		1
Relapse			
No	<.001	0.2	0.1 to 0.3
Yes	1		1
Parity			
>4	.058	0.5	0.3 to 1.02
<4	1		1
Hypertension			
Yes	.02	1.9	1.09 to 3.7
No	1		1
ECOG			
≥1	<.001	2.5	1.4 to 4.8
0	1		1
Smoking			
No	<.001	0.12	0.02 to 0.5
Yes	1		1
Trastuzumab use			
No	.001	3.9	1.4 to 4.2
Yes	1		1
Adjuvant chemotherapy			
No	.04	2.17	1.06 to 4.5
Yes			
Hormonal therapy			
No	.08	1.9	0.9 to 2.9
Yes	1		1
Stage			
III	.002	4	2.1 to 8.1
II			
Insurance status			
Yes	.003	0.4	0.2 to 0.7
No		1	1

Abbreviations: cHR, crude hazard ratio; ECOG, Eastern Cooperative Oncology Group.

According to the Schoenfeld residual test, the proportional hazards assumption was violated (*P* = .0008). To address this, a Cox regression analysis with the addition of a time-dependent covariate was performed. The study included 169 patients and 57 events. The concordance rate was 0.788. The effect of trastuzumab use on mortality changed by 5% from a follow-up period of <6 months to more than 6 months.

Trastuzumab, when not used, was associated with a 13.95-fold increased risk of mortality (adjusted odds ratio [aOR], 13.95, *P* < .001). Patients with advanced-stage cancer had a 3.1-fold higher risk of mortality (aOR, 3.1, *P* = .001). Nonsmoking status was associated with a 95% lower risk of mortality (aOR, 0.05, *P* < .001). Postmenopausal patients had a 57% lower mortality risk compared with premenopausal patients (aOR, 0.43, *P* = .004). By contrast, health insurance status did not significantly affect mortality (aOR, 0.94, *P* = .87; Table 3).

**TABLE 3:** A Summary of Multivariate Analysis (Cox regression analysis with the addition of a time-dependent covariate)

Variable	Multivariate analysis		
	*P*	aHR	95% CI
Menopausal status			
Postmenopausal	.004	0.43	0.25 to 0.77
Premenopausal	1		
Smoking			
No	<.001	0.05	0.01 to 0.27
Yes	1		
Trastuzumab use			
No	<.001	13.9	3.83 to 50.76
Yes	1		
Trastuzumab use (TT)	.03	0.95	0.91 to 0.99
Stage			
III	.001	2.9	1.54 to 6.25
II	11		
Insurance status			
Yes	.94	0.9	0.49 to 1.82
No	1		

Abbreviation: aHR, adjusted hazard ratio.

## DISCUSSION

In this study, 58% of participants presented with advanced-stage BC. The majority of BC cases in Tanzania are diagnosed at advanced stages, which limits treatment options and results.^13-16^ Key factors include reliance on traditional healing, poor health-seeking behavior, limited awareness, and economic or geographical barriers.^13-16^ A similar pattern is seen in other LMICs. A study by Singh et al^17^ from resource-constrained North India showed that 50% of patients with HER2+BC were diagnosed at stage III. BC studies in different parts of Africa have yielded similar results; in Ethiopia, 64% of women were diagnosed at advanced stages, influenced by limited knowledge of the disease and use of traditional medication (Gebremariam et al, 2022).^18^ In Ghana and Uganda, the majority of women presented with late-stage BC, driven by factors such as lack of awareness, poverty, and reliance on traditional healing, deficient or absent screening programs, and lack of social support.^19,20^ These studies highlight the need for targeted interventions to improve early detection, public awareness, and health care accessibility in these regions.

About half of the study participants (50.6%) received treatment after paying out of pocket, with less than half (44.2%) being able to receive trastuzumab. Similar findings were observed in Nigeria, where out-of-pocket expenditure for BC treatment was estimated to be 68%, and the mean out-of-pocket cost of trastuzumab, including direct and indirect expenses, was $6,568 US dollars (USD; $2,766 USD) for 7.5 doses.^21^ Higher rates of out-of-pocket expenditure may lead to catastrophic health expenditures for patients.^21^ A study from Kenya highlighted the high out-of-pocket costs of trastuzumab for patients with HER2+BC (HER2–BC), where the drug costs 111,244 KES (approximately $990 USD). Only 33.6% of patients completed the full treatment, and many received fewer cycles because of financial constraints.^22^ This study recommended negotiating drug subsidies and adjusting National Health Insurance Fund policies to enhance accessibility. The extent to which health systems protect individuals from financial shock or impoverishment caused by illness is a key indicator of their performance.^23^ In a study by Knapp et al^21^ in Nigeria, approximately 86%-95% of households experience financial catastrophe because of one member being diagnosed with BC. Based on these results, estimating the out-of-pocket costs associated with receiving trastuzumab and determining how these costs affect access to this life-saving treatment are necessary.

Most participants in the index were unemployed (76.9%) or had a lower level of education (79.3%). Our study showed that employed, educated women were more likely to receive trastuzumab compared with uneducated and unemployed women. This finding is consistent with a study in northern India, where patients receiving trastuzumab were predominantly from upper- or middle-class socioeconomic backgrounds and were well-educated, with 75% of recipients having higher education compared with 28.2% of nonrecipients (*P* = .0009).^17^ Similar trends are found even in developed nations; one study by Liu et al^25^ in China revealed that individuals with lower educational attainment or lower-paying jobs were more likely to present with later-stage tumors and had lower rates of receiving comprehensive treatments, including trastuzumab. By contrast, another study by Li et al^24^ showed that in resource-limited regions, more than 40% of patients never received trastuzumab, compared with <10% in resource-rich areas.^24,25^

The first cost-effectiveness analysis of HER-2 targeted therapy in SSA, conducted by Gershon et al,^26^ suggested that trastuzumab is not cost-effective in low-income settings. According to the Breast Health Global Initiative, trastuzumab is only considered appropriate in high-income settings.^27^ The high cost of trastuzumab, compounded by limited health insurance coverage, presents a significant barrier to treatment access in Tanzania. Studies confirm that the affordability of trastuzumab remains a significant challenge in low-income settings, where most patients are from low-income households.^21^ Insurance coverage proved to be critical as insured patients had better access to trastuzumab than those paying out of pocket. These findings align with those in Ghana, where insured patients with BC experienced significantly improved survival rates.^22^ Introducing biosimilars could help reduce costs and enhance access, as demonstrated in India, where the availability of biosimilars lowered treatment costs from $30,600 USD to $11,300 USD, making it accessible even to uninsured patients.^28-30^ Expanding insurance coverage and reimbursement policies is crucial for mitigating socioeconomic disparities in BC treatment, as seen in China's insurance reforms, which increased access to trastuzumab and reduced out-of-pocket costs.^31-33^ Currently, Tanzanian patients shoulder 58% of the cost of essential cancer drugs, compared with just 1.8% in high-income countries.^33^

Our study found that trastuzumab was more commonly used in adjuvant chemotherapy than NACT (69.0% *v* 41.7%). Although neoadjuvant trastuzumab has shown improved pathologic response rates,^34,35^ limited evidence of survival benefits and concerns about cardiotoxicity in patients with low functional status may explain the preference for adjuvant use.^36^ In addition, neoadjuvant therapy in Tanzania is frequently reserved for advanced-stage cases where surgical outcomes are less predictable. A study by Li et al^24^ had similar findings, whereby most patients received trastuzumab in adjuvant rather than neoadjuvant settings (30.5% *v* 18.3%). Another study from Brazil showed that the majority of patients receiving neoadjuvant trastuzumab had advanced BC at diagnosis, unlike those who received adjuvant trastuzumab, with those receiving adjuvant trastuzumab having better 3- (97% *v* 79%) and 5-year OS rates (92% *v* 79%) compared with the neoadjuvant group.^37^

This cohort's 3-year OS rate of 57% and median survival time of 72 months underscore the need for extended trastuzumab therapy to improve outcomes. Patients receiving more than eight cycles of trastuzumab showed better survival rates, particularly among patients with ER-negative HER2+BC.^38-40^ A study by Ansaripour et al^41^ showed that shorter durations of adjuvant trastuzumab have comparable 4-year disease-free survival with standard 1-year therapy and can be an alternative adjuvant treatment option in resource-limited settings. However, the study did not analyze patients based on their hormonal status, and the majority had early-stage, hormone-positive breast cancer.^41^ The PHARE trial showed that 6 months of trastuzumab was less effective than 12 months, regardless of hormone receptor status. However, there were some nuances: Hormone receptor–negative patients (who generally have more aggressive disease) benefited more significantly from the full 12-month treatment compared with the 6-month regimen.^42^ Findings from the PERSEPHONE trial suggested that a shorter 6-month trastuzumab regimen is noninferior to a 12-month regimen for early BC, reducing costs and toxicity.^43^ This regimen has already been adopted in resource-limited settings, such as South Africa, demonstrating that shorter trastuzumab treatments can be a viable option without compromising outcomes.^41,44^ However, patients with ER-negative HER2+ BC may still benefit from the entire 12-month course, which is preferable when feasible.

The study also highlights a disparity in median survival times based on insurance status, reinforcing that access to health insurance positively influences BC treatment and survival.^45,46^ A study by Ko et al^47^ underscores this by showing that uninsured patients face poorer survival outcomes and are often diagnosed at later stages. With only 15% of Tanzanians insured, there are significant health coverage disparities,^10^ which highlights the need for policy reforms to improve access to cancer treatment and reduce out-of-pocket expenses.

Higher mortality rates observed in premenopausal women may reflect the aggressive nature of BC in younger patients. Consistent with other research studies, our findings suggest that younger patients with BC, often diagnosed with more aggressive cancers, face greater mortality risks.^48-50^ Increased awareness and targeted interventions could help mitigate these risks.

Based on our data, smoking was associated with higher mortality, which is in line with a wealth of evidence showing the detrimental effects of smoking on cancer prognosis and OS.^51-53^ Based on a study by Raghavendra et al,^51^ quitting smoking after a breast cancer diagnosis is linked to substantially improved survival outcomes. Smoking has been shown to undermine cancer treatment effectiveness by influencing the immune tumor microenvironment, which, in turn, affects how the body responds to chemotherapy.^54^ It elevates the risks of late side effects post-therapy, increases the risk of distant metastasis and recurrence, and drives up health care costs.^37,38^ Interestingly, the benefits of smoking cessation hold regardless of other prognostic factors. These findings highlight the critical importance of smoking cessation support as part of comprehensive breast cancer care. Oncologists should routinely screen for smoking and connect patients to impactful cessation services.

The study has several limitations that need to be taken into consideration. Although it was performed at two prominent tertiary institutions in Tanzania, which are central to cancer treatment in the country, the sample size of 169 patients may limit the statistical power and the ability to identify significant differences among subgroups. The retrospective nature of this study might have introduced information bias as it relies on potentially incomplete medical records. The retrospective nature made it difficult to use a standardized method to evaluate participants' financial status in depth, which limited our understanding of how socioeconomic factors can influence treatment access and adherence.

Despite these limitations, the study's findings are likely to be relevant to other sub-Saharan African countries facing similar socioeconomic challenges, such as widespread poverty, limited access to health insurance, low educational attainment, and restricted health care services. Given variations in local health care systems, it is essential to exercise caution when applying these results to diverse contexts. Future multicenter and prospective studies incorporating standardized socioeconomic assessments are crucial for validating these findings and achieving a more comprehensive understanding of treatment outcomes in resource-constrained settings. Furthermore, a qualitative study is needed to assess the barriers and facilitators of incorporating trastuzumab in resource-limited settings, which could be used to design implementation strategies to enhance access to this drug.

In conclusion, this study highlights critical challenges in breast cancer treatment in Tanzania, particularly around access to trastuzumab. High rates of advanced-stage diagnosis, socioeconomic barriers, limited insurance coverage, and high medication costs restrict access to this life-saving therapy. Trastuzumab usage was notably higher in adjuvant settings, with those receiving more than eight cycles showing improved survival outcomes, especially among patients with ER-negative HER2+BC. The 3-year survival rate of 57% underscores the potential benefit of expanded trastuzumab availability, which could be facilitated through policy reforms that increase insurance coverage and enable access to cost-effective biosimilars. A shorter-duration trastuzumab regimen is cost-effective in other resource-limited settings and could also provide a viable treatment option to reduce costs without compromising efficacy. Addressing the cost and accessibility challenges through policy interventions, public health education, and insurance reform is essential to improving breast cancer survival outcomes in Tanzania.

## Data Availability

The data set used and analyzed during the current study is available from the corresponding author upon reasonable request.
